# The Educational Impact of Web-Based, Faculty-Led Continuing Medical Education Programs in Type 2 Diabetes: A Survey Study to Analyze Changes in Knowledge, Competence, and Performance of Health Care Professionals

**DOI:** 10.2196/40520

**Published:** 2022-10-14

**Authors:** Stewart B Harris, Shannon Idzik, Adriano Boasso, Sola Quasheba Neunie, Alexander Daniel Noble, Helen Elaine Such, Joanna Van

**Affiliations:** 1 Schulich School of Medicine and Dentistry Western University London, ON Canada; 2 University of Maryland School of Nursing Baltimore, MD United States; 3 touchIME Stockport United Kingdom; 4 University Clinical Investigators (d.b.a. Diabetes Research Center) Tustin, CA United States

**Keywords:** clinical case, competence, continuing medical education, knowledge, multidisciplinary team, web-based education, performance, type 2 diabetes

## Abstract

**Background:**

The treatment landscape for type 2 diabetes (T2D) is continually evolving; therefore, ongoing education of health care professionals (HCPs) is essential. There is growing interest in measuring the impact of educational activities, such as through use of the Moore framework; however, data on the benefits of continuing medical education (CME) in the management of T2D remain limited.

**Objective:**

This study aimed to evaluate HCP satisfaction; measure improvements in knowledge, competence*,* and performance following short, case-based, multidisciplinary web-based CME activities*;* and identify the remaining educational gaps.

**Methods:**

Two faculty-led, CME-accredited, web-based educational activities on T2D and obesity, touchIN CONVERSATION and touch MultiDisciplinary Team, were developed and made available on a free-to-access medical education website. Each activity comprised 3 videos lasting 10 to 15 minutes, which addressed learning objectives developed based on a review of published literature and faculty feedback. Participant satisfaction (Moore level 2) was evaluated using a postactivity questionnaire. For both activities, changes in knowledge and competence (Moore levels 3 and 4) were assessed using questionnaires completed by representative HCPs before or after participation in the activities. A second set of HCPs completed a questionnaire before and after engaging in activities that assessed changes in self-reported performance (Moore level 5).

**Results:**

Each activity was viewed by approximately 6000 participants within 6 months. The participants expressed high levels of satisfaction (>80%) with both activities. Statistically significant improvements from baseline in knowledge and competence were reported following participation in touchIN CONVERSATION (mean score, SD before vs after activity: 4.36, 1.40 vs 5.42, 1.37; *P*<.001), with the proportion of learners answering at least six of 7 questions correctly, increasing from 22% (11/50) to 60% (30/50). A nonsignificant improvement in knowledge and competence was observed following participation in touch MultiDisciplinary Team (mean score, SD 4.36, 1.24 vs 4.58, 1.07; *P*=.35); however, baseline knowledge and competence were relatively high, where 80% of the respondents (40/50) answered at least four of 6 questions correctly. A significant improvement in HCP self-reported performance was observed in a combined analysis of both activities (mean score, SD 2.65, 1.32 vs 3.15, 1.26; *P*=.03), with the proportion of learners selecting the answer representing the best clinical option for all 4 questions increasing from 32% (11/34) to 59% (20/34) after the activity. Several unmet educational needs were self-reported or identified from the analysis of incorrectly answered questions, including setting individualized glycemic targets and the potential benefits of sodium-glucose cotransporter 2 inhibitor therapies.

**Conclusions:**

Short, case-based, web-based CME activities designed for HCPs to fit their clinical schedules achieved improvements in knowledge, competence, and self-reported performance in T2D management. Ongoing educational needs identified included setting individualized glycemic targets and the potential benefits of sodium-glucose cotransporter 2 inhibitor therapies.

## Introduction

### Epidemiology and Burden of Type 2 Diabetes

Diabetes is a major public health concern worldwide. In 2021, it was estimated to affect 537 million adults (9.8% of the world’s population) and was responsible for 6.7 million deaths [1]. Type 2 diabetes (T2D) is the most common type of diabetes, accounting for more than 90% of all cases worldwide, and is often associated with lifestyle factors, such as an unhealthy diet and obesity [1]. It is well established that reducing levels of glycated hemoglobin (HbA_1c_) in patients with diabetes can delay the onset and progression of microvascular and macrovascular complications [2,3]. An HbA_1c_ <7% is recognized by both the American Diabetes Association and the European Association for the Study of Diabetes as an appropriate glycemic target [4,5], although the proportion of patients with T2D who achieve HbA_1c_ <7% varies from approximately 20% to 50% in different regions of the world [6,7]. Thus, there remains a need to achieve optimal glycemic control in patients with T2D [6]. However, diabetes management has become increasingly complex for health care professionals (HCPs) owing to multiple medication classes and treatment combinations, the need to avoid hypoglycemia or hyperglycemia, multiple medical device options, and the need to facilitate patients’ lifestyles [8]. This multiplicity of treatment options, combined with the need to manage the risk of complications in patients with T2D, underscores the need for HCP education to ensure optimal patient management according to the most recent guidelines and evidence-based practice [9,10]. T2D management has also evolved from a glucocentric approach aimed at achieving glycemic control to a holistic approach aimed at preventing complications and improving quality of life, with a 2018 consensus report from the American Diabetes Association and European Association for the Study of Diabetes, highlighting the importance of person-centered care [5,11]. Specifically, it is now recommended that the selection of add-on therapy after metformin should be based on factors other than just HbA_1c_, and decision-making should also take into account the presence of comorbidities such as atherosclerotic cardiovascular disease, heart failure, and chronic kidney disease, as well as the patient’s clinical characteristics, risks for side effects, and socioeconomic factors [11]. This person-centered approach to T2D management is particularly relevant to primary care providers, such as family physicians, internists, nurse practitioners, and physician assistants. Thus, given the increase in the prevalence of T2D in countries such as the United States, primary care providers play a key role in ensuring that patients who do not require specialist care remain at low risk of complications and comorbidities and can be effectively managed in a primary care setting [11]. The focus of primary care providers on the prevention of T2D progression or worsening also makes them well placed to lead a person-centered approach to diabetes care with the aim of achieving both good glycemic control and reducing the risk of complications [11].

### Education in T2D Management

As the treatment landscape and guidelines for T2D are continually evolving, innovative educational activities are required to ensure that HCPs remain up to date with clinical developments in the management of the disease. In addition, a multidisciplinary and person-centered approach is recommended for the management of patients with diabetes [12]. In support of this, a position statement published by the Insights for Diabetes Excellence, Access, and Learning Group in 2020 emphasized the need for responsive and effective HCP education to meet the increasing needs for diversity, specialism, cultural competence, advancing practice, and person-centeredness in diabetes care delivery [13]. They also highlighted the importance of proactive rather than reactive diabetes care to avoid therapeutic inertia in timely treatment intensification [13]. Many activities focusing on HCP education in the management of T2D have been developed. Although some peer-reviewed publications have described outcomes from educational activities in diabetes, these are highly heterogeneous, may include patients with type 1 diabetes, and may include a range of activities and a variety of end points [9,10,14-20].

### Medical Education for HCPs

Traditionally, ongoing medical education for HCPs worldwide involves live symposia, face-to-face workshops, and training events. However, for many HCPs, these can be cost and time prohibitive [21,22]. As an alternative, web-based distance learning offers many advantages, including ease of access, ability to take a course from any location, lower cost of delivery, and availability at any time [21,22]. With the onset of the COVID-19 pandemic, the need to digitize medical education programs became even more urgent to ensure that HCPs had continued access to education in the absence of face-to-face events [23]. Several studies and commentaries published during this time illustrated that web-based events can be effective and can reduce barriers to access [24-26]. Effective web-based educational activities depend on many factors, including well-designed course content and well-prepared and fully supported instructors [27]. Education should also be in the context of patient care, answer HCP questions, and be directly applicable to their work [28]. Precanvassing the target audience, for example, asking potential learners to provide specific questions or cases that they would like the activity to cover, can be valuable, as it theoretically allows learners to become more vested in the activity by contributing questions on their own key educational areas of interest. Involving learners in the identification of educational gaps is a well-known tool for designing educational activities that can effectively impact theoretical and practical knowledge [29]. Precanvassing the audience allows us to involve learners in activity development and encourages participation and engagement. The value of adopting surveys of the target audience to identify needs and shape educational programs has been previously demonstrated [30]. Delivering content using an engaging format may be particularly important in the digital age, given the competing demands for individuals’ attention [31].

The need for interdisciplinary medical education has arisen because medicine has become increasingly specialized in recent decades; this can be effectively met by various specialties presenting content to a multidisciplinary audience of HCPs [32]. With studies demonstrating the high use of freely available medical education by a multidisciplinary audience, it is important for medical education providers to address this need by providing interdisciplinary programs [32]. The World Health Organization has also highlighted that medical education providers and programs should deliver education that helps HCPs acquire wide-ranging competencies, including multidisciplinary patient care [33].

### Assessing the Impact of Education

For many years, HCPs only had to provide documentation of attendance at educational activities to qualify for certification by their professional associations or the reregistration of their medical licenses [34]. However, there is growing recognition of the need to assess the impact of continuing medical education (CME) on HCP performance and health outcomes [34]. The Accreditation Council for Continuing Medical Education (ACCME) now requires CME providers to demonstrate changes in learner competence, performance, or patient outcomes because of the program [35]. Similarly, the American Nurses Credentialing Center requires that an accredited provider measures the impact of its educational activities in relation to improving the knowledge, skills, and practices of registered nurses [36].

In 2009, Moore et al [34] developed an expanded 7-level framework for planning and assessing the outcomes of a CME program. Level 1 measures the number of HCPs who participate in an activity, and level 2 measures the extent to which they are satisfied with it, using a questionnaire completed after the activity. Levels 3 and 4 measure knowledge and competence, respectively. Knowledge can be assessed either objectively through pre- and postactivity tests or subjectively through self-reports of knowledge gain. Competence can be assessed either objectively by observation in an educational setting or subjectively by self-reporting competence or intention to change. Level 5 measures performance and can be objectively assessed through performance in a patient care setting or subjectively assessed through self-reporting of performance. Levels 6 and 7 measure the degree to which education can improve the health status of patients or a community of patients through an analysis of health status measures in patient charts or databases or of epidemiological data [34]. Moore levels have been widely used to measure the outcomes of educational programs and have been included in many consensus documents and best practice recommendations [37].

In this study, we developed and implemented 2 faculty-led, CME-accredited, web-based educational activities on T2D and obesity and analyzed the educational outcomes up to Moore level 5. The objectives of this analysis were to (1) evaluate the learners’ satisfaction with the educational activities, as well as the changes in knowledge, competence, and performance that were achieved following their implementation and (2) identify the remaining educational gaps in the clinical management of T2D and obesity.

## Methods

### Educational Activities

Educational gaps were identified at the start of activity development, in March 2021, by touch Independent Medical Education (touchIME), a provider of independent medical education for the global HCP community. The identification process included a thorough review of the relevant published literature on T2D and feedback from expert faculty specializing in diabetes care and research.

The expert faculty and patient faculty member were identified and recruited by the medical directors at touchIME. The expert faculty was identified through searches of the literature, relevant congress websites, and web-based educational videos, for diabetes experts with an established background in diabetes research and clinical practice. The Patient faculty member was identified through searches for videos or blogs detailing the firsthand experience of a patient with T2D and obesity. Recruitment was conducted by email invitation, which included details of the proposed activity. Conflict of interest statements from all faculty participants were gathered during the recruitment stage. All the expert faculty members involved in the educational activities are authors of this manuscript or mentioned in the acknowledgments.

Learning objectives were designed based on the educational gaps, and 2 faculty-led, web-based, CME-accredited activities were developed, each comprising 3 recorded 10- to 15-minute videos that addressed the learning objectives (Multimedia Appendix 1). The identified educational gaps and corresponding learning objectives for touchIN CONVERSATION and touch MultiDisciplinary Team (touchMDT) are listed in Table S1 in Multimedia Appendix 2. Both activities were recorded remotely using a web-based video conferencing platform and were made available to the HCPs for a maximum of 24 months after launch.

The first activity, touchIN CONVERSATION, featured an endocrinologist and a diabetes specialist and focused on the management of specific patient cases in the clinic. The learning objectives were to (1) evaluate the unmet need for achieving glycemic control and the associated reasons, (2) decide how to apply individualized glycemic targets according to patient characteristics, and (3) choose appropriate treatments with properties relevant to the individual patient to help achieve glycemic control. For the activity to be immediately relevant to participants’ daily practice, the target audience was precanvassed for questions related to specific patient cases. Precanvassing was carried out by touchIME starting 4 weeks before the videos were recorded, whereby HCPs were invited to submit questions based on patient cases on the following key topics: (1) challenges faced in achieving glycemic control, (2) applying individualized glycemic targets according to patient characteristics, and (3) treatment choices for achieving glycemic targets safely. Canvassing through social media took place using Facebook, LinkedIn, and Twitter, with announcements targeting relevant HCPs, identified using keywords in their profiles linked to diabetes and endocrinology. Canvassing through organic social media involved nontargeted announcements on the same 3 channels, as well as on touchENDOCRINOLOGY and touchCARDIO websites. In addition, an announcement was sent directly to 11,586 HCPs who had subscribed to emails from the touchENDOCRINOLOGY and touchCARDIO sites. No financial incentives were provided to submit the questions. For each video, 3 questions were developed by the faculty for discussion. The questions were based on the precanvassing and learning objectives of the activity.

The second activity, a touchMDT, featured an endocrinologist, a primary care physician (PCP), a nurse specializing in diabetes, and a patient with T2D. This study focused on the relationship between T2D and obesity, and the learning objectives were to (1) describe the relationship between T2D and obesity, (2) predict the beneficial effects of weight loss with glucagon-like peptide-1 (GLP-1) receptor agonist (GLP-1 RA)–based therapy and/or sodium-glucose cotransporter 2 inhibitor (SGLT2i) therapy on outcomes in patients with T2D and obesity, and (3) perform appropriate selection of antihyperglycemic therapy with weight loss benefits for patients with T2D and obesity. Each of the three 10-minute discussions involved a different combination of clinicians and the patient and was based on 3 or 4 discussion points focused on the practical management of patients with T2D and obesity from both the clinicians’ and the patient’s perspectives.

Both activities are available as free to access on the touchENDOCRINOLOGY website (Multimedia Appendix 1) [38], a web-based HCP education community, from October 2021 to October 2023. The maximum 24-month viewing period was set in accordance with ACCME requirements. To reach a global target audience of HCPs specializing in diabetes, endocrinology, or primary care, a combination of communication channels was used, including emails—using touchMAIL, touchIME’s proprietary software—to touchENDOCRINOLOGY subscribers within the first 12 weeks and then 6 months after activity launch; medical society partnerships (website publicity) throughout the lifetime of activity; and HCP-targeted social media announcements on Facebook, LinkedIn, and Twitter throughout the lifetime of activity. The announcements on these social media platforms were paid. No financial incentives were provided to participate in this activity.

CME accreditation for both activities was provided by the University of South Florida Health, which is accredited as a provider of continuing professional development by the ACCME and the American Nurses Credentialing Center.

### Assessment of Educational Outcomes

Outcomes for both activities were assessed according to the Moore expanded outcomes framework (levels 1-5) [34]. For levels 1 to 4 (participation, satisfaction, knowledge and competence), the outcomes were assessed for the 2 activities independently. For level 5 (performance), outcomes were assessed for the 2 activities combined to account for the overlap in content, learning objectives (ie, treatment selection and intensification for patients with T2D and overweight or obesity), and target audience.

Level 1 was assessed over the first 6 months after launch as 2 variables: the number of participants who engaged in the activity and the average time spent by participants viewing the videos. Google Analytics was used to capture geolocation, participant numbers, and the overall average time HCPs spent on the activity. Data on specialty and the country from which participants connected were collected from HCPs who viewed the activity using their touchENDOCRINOLOGY account and from learners who completed the level 3 and level 4 outcome questionnaires.

Levels 2 to 5 were assessed using the outcome questionnaires. To avoid bias, all data from the level 2 to level 5 questionnaires were collected by an independent third-party vendor (nuaxia Limited) that was not involved in the development of the activities. A target audience was specified for fielding the questionnaires so that the sample was taken from relevant respondents (HCPs who completed the preactivity questionnaire) and learners (HCPs who participated in the activity and completed the postactivity questionnaire). Financial incentives were provided by nuaxia Limited for the HCPs to complete the questionnaires. For both activities, the target audiences were predefined by specialty (diabetologists, endocrinologists, and primary care specialists) and country (France, Germany, Italy, Spain, and the United States). A database of 203,744 HCPs was sampled based on a predefined target audience. To avoid any pre-exposure bias and obtain a statistically representative sample size, data were collected using an independent sample model both before and after the launch of each activity. All questionnaires were fielded to the database and then closed once a prespecified number of HCPs responded. Levels 3 and 4 are assessed using a single questionnaire. Preactivity scores were obtained by fielding this questionnaire 1 to 2 weeks before launch (to ensure that the sample was from HCPs who had not interacted with the activity) and were closed after 50 respondents had completed it. Postactivity scores were obtained by fielding this questionnaire to another set of HCPs immediately after the launch and closed after 50 learners responded. The level 2 questionnaire assessing satisfaction with the activities was included with the level 3 and level 4 questionnaire that was fielded after the activity. For level 5, the questionnaire was fielded 1 to 2 weeks before the launch—to a different set of HCPs to those who answered the level 3 and level 4 questionnaires—and was closed after 50 respondents had completed it. At 26 weeks after the launch, the level 5 questionnaire was administered to the same 50 learners who had responded before the activity; of these, 34 (68%) responded to the postactivity fielding, and data are presented as paired samples from these 34 learners only. For levels 2 to 5, the learners who responded to the postactivity questionnaires viewed the activity as part of the questionnaire process.

The level 2 satisfaction questionnaire included the following 5 statements that were to be scored using a 1- to 5-point Likert scale (where 5 is the highest satisfaction): this activity was of high quality, this activity met the stated learning objectives, the presenters were knowledgeable and effective, the activity contained content relevant to my clinical practice, and the information presented is likely the help change my management strategies in this therapeutic area. Levels 3 and 4 (knowledge and competence) and level 5 (performance) were assessed using questionnaires developed by the medical directors at touchIME and approved for scientific and medical accuracy by the faculty (Textbox 1). Satisfaction data were collected from participants immediately after engaging in the activity and before answering the level 3 and level 4 questionnaires. The level 3 and level 4 questionnaires comprised 7 questions for touchIN CONVERSATION (Table S2 in Multimedia Appendix 2) and 6 questions for touchMDT (Table S3 in Multimedia Appendix 2). All the questions were multiple-choice, with 3 to 4 possible answers, of which only one was correct. Data were analyzed for the overall participant groups and in subgroups defined by country, specialty, and years of experience. The level 5 questionnaire is a subjective assessment based on self-reported change in performance. It included 4 multiple-choice questions with 4 possible answers. All answers were plausible, but one was the best possible clinical option (Table S4 in Multimedia Appendix 2).

**Topics included in the level 3, level 4, and level 5 outcome questionnaires.**

**Levels 3 and 4**

To assess levels 3 and 4, separate questionnaires were developed for touchIN CONVERSATION and touch MultiDisciplinary Team activities

- touchIN CONVERSATION
  Factors contributing to clinical inertia
  Achieving glycemic control in nonadherent patients
  Selecting individualized glycemic targets and add-on therapies
  Emerging therapies for patients with type 2 diabetes (T2D) and overweight or obesity
- touchMDT (touch MultiDisciplinary Team)
  Mechanisms linking obesity to T2D
  Benefits of weight loss for T2D prevention
  Clinical benefits of glucagon-like peptide-1 receptor agonists (GLP-1 RAs) and sodium-glucose cotransporter-2 inhibitors (SGLT2is)
  Treatment intensification after a GLP-1 RA or metformin

**Level 5**

To assess level 5, a single questionnaire was sent to the respondents of both activities

- Appropriate second-line treatment selection for patients with T2D and overweight or obesity
- Eligibility criteria for treatment with a GLP-1 RA
- Outcomes expected for patients treated with an SGLT2i
- Treatment intensification in patients with T2D, obesity, and atherosclerotic cardiovascular disease, who have not achieved their glycemic target

### Intention to Change Practice

To assess the impact of the educational activities on HCPs’ willingness to change their clinical practice, learners who took part in the level 2 to level 5 questionnaires after participating in the activities were asked the following multiple-choice question: “As a result of your participation in this session, will you make a change in your practice?” There were 5 possible mutually exclusive responses: yes, uncertain—more education needed, uncertain—practical limitations, no—more education needed, and no—practical limitations.

### Identification of Remaining Educational Gaps

To collect information on the learners’ perspective on the need for further education in the management of T2D, those who completed the level 2 to level 5 questionnaires after the activity were asked the question, “What do you think is the most important unmet educational need in this therapy area?” They were required to rank 4 predefined potential educational gaps (12 in total over the 3 questionnaires) by importance. Potential educational gaps were drafted by the medical directors at touchIME, with input from the faculty on the respective activities, and were included at the end of the questionnaires after the multiple-choice questions. The results were analyzed using a single transferable vote system. In the first round of voting, all first-choice votes were counted to determine the most important educational gap for the participants; in the second round, all second-choice votes were counted to determine the second most important educational gap. Any first-choice vote, not from the winning option in the first round, was also counted in the second round. The voting rounds continued until all options were placed in order. In addition, questions in the level 3 and level 4 questionnaires that were answered incorrectly by ≥40% of learners after completion of the activity were identified as outstanding educational gaps.

### Statistical Analysis

Data were analyzed using SPSS Statistics (version 28.0.1; IBM Corp). On the basis of target population of learners and the sample size, a statistical power calculation was used to determine the number of respondents (N=50) and learners (N=50) required to detect a statistically significant difference between surveys conducted before and after the activity, with a margin of error of approximately 10% for both touchIN CONVERSATION and touchMDT. For the satisfaction (level 2) questionnaire, the mean scores were calculated for the individual questions, and an overall satisfaction score was calculated as the average across all satisfaction fields, with a maximum possible satisfaction score of 5 points out of 5. For the knowledge and competence (levels 3 and 4) analysis, the mean and median numbers of correct answers were calculated for both the pre- and postactivity data sets, and the results were compared using an independent sample 2-tailed *t* test. To analyze the results by country, specialty, and experience, 2-way ANOVA was used. Individual questions were first analyzed using a paired sample *t* test and then using 1-way ANOVA. Data collection for performance (level 5) was performed using a matched sampling method. Pre- and postactivity data were compared using a paired sample *t* test. Country, specialty, and experience analyses were conducted using the same methods as for levels 3 and 4 using 2-way ANOVAs.

### Ethics Approval

The faculty for touchIN CONVERSATION and touchMDT consented to the necessary use, distribution, and reproduction of their contribution to the activities and assigned the entire copyright and all other intellectual property rights existing in their contributions to touchIME. According to the European Union General Data Protection Regulation [39], HCPs who responded to the outcome questionnaires were informed before their input that, as with all research, their identity and personal data were strictly confidential and would not be revealed without their explicit further consent. This study did not report experiments on human participants; therefore, institutional review board approval and informed consent were not applicable.

## Results

### Assessment of Educational Activities

#### Level 1—Participation

Data collected between 6 and 7 months after launch showed that 6759 and 5998 participants had engaged with the touchIN CONVERSATION and touchMDT activities, respectively. The average length of participation was 8.50 minutes for the touchIN CONVERSATION and 13.09 minutes for the touchMDT (Table 1). For both activities, most participants specialized in endocrinology or diabetes, with only a small proportion working in primary care. Most participants were physicians (8869/12,757, 69.5%, for both activities combined), with the remainder being either nurse practitioners (2551/12,757, 20%) or physician assistants (1335/12,757, 10.5%). Participants from 25 countries engaged in each activity. The largest proportion of HCPs who engaged in the touchIN CONVERSATION activity was based in the United States, followed by the Philippines and Italy. The largest proportion of HCPs who participated in the touchMDT activity were based in the United Kingdom, followed by the United States, Portugal, and Italy. All other countries were represented by fewer than 10% of participants for each activity (Table 1).

**Table 1:** Engagement results and demographics of participants in the touchIN CONVERSATION and touch MultiDisciplinary Team (touchMDT) activities^a^.

		touchIN CONVERSATION		touchMDT
Participant engagement, n		6759		5998
Countries reached, n		25		25
Length of participation (minutes), mean (SD)		8.50		13.09
**Specialty, n (%)**				
	Endocrinology	3109 (46.00)	3178 (52.98)	
	Diabetes	2500 (36.99)	1979 (32.99)	
	Primary care	1149 (17.00)	839 (13.99)	
	Not reported	1 (0.01)	2 (0.03)	
**Role, n (%)**				
	Physician	4731 (70.00)	4138 (68.99)	
	Physician assistant	676 (10.00)	659 (10.99)	
	Nurse practitioner	1352 (20.00)	1199 (19.99)	
**Country^b^, n (%)**				
	United States	2064 (30.54)	1319 (21.99)	
	Philippines	1323 (19.57)	N/A^c^	
	Italy	872 (12.90)	574 (9.57)	
	India	507 (7.50)	100 (1.67)	
	Bangladesh	466 (6.89)	N/A	
	Australia	242 (3.58)	148 (2.47)	
	Pakistan	222 (3.28)	N/A	
	United Kingdom	161 (2.38)	1470 (24.51)	
	Spain	137 (2.03)	338 (5.64)	
	Ireland	107 (1.58)	N/A	
	Portugal	N/A	657 (10.95)	
	Canada	N/A	280 (4.67)	
	Netherlands	N/A	149 (2.48)	
	Mexico	N/A	116 (1.93)	

^a^Data collected on April 22, 2022, and at 203 and 190 days after the launch of touchIN CONVERSATION and touchMDT, respectively.

^b^Country where the participant was based at the time of completing the activity. Data are reported for countries represented by ≥2% of the participants for at least one activity.

^c^N/A: not applicable.

#### Level 2—Satisfaction

The overall satisfaction scores were 84% (4.2/5) for touchIN CONVERSATION and 82% (4.2/5) for touchMDT. For touchIN CONVERSATION and touchMDT, respectively, satisfaction scores of 4.2 and 4.1 for the quality of the activity, 4.2 and 4.2 for meeting the stated learning objectives, 4.3 and 4.3 for the knowledge and effectiveness of the presenters, 4.4 and 4.1 for relevance to clinical practice, and 4.0 and 3.8 for impact on management strategies were achieved out of a maximum score of 5.0.

#### Levels 3 and 4—Knowledge and Competence

**Figure 1:**
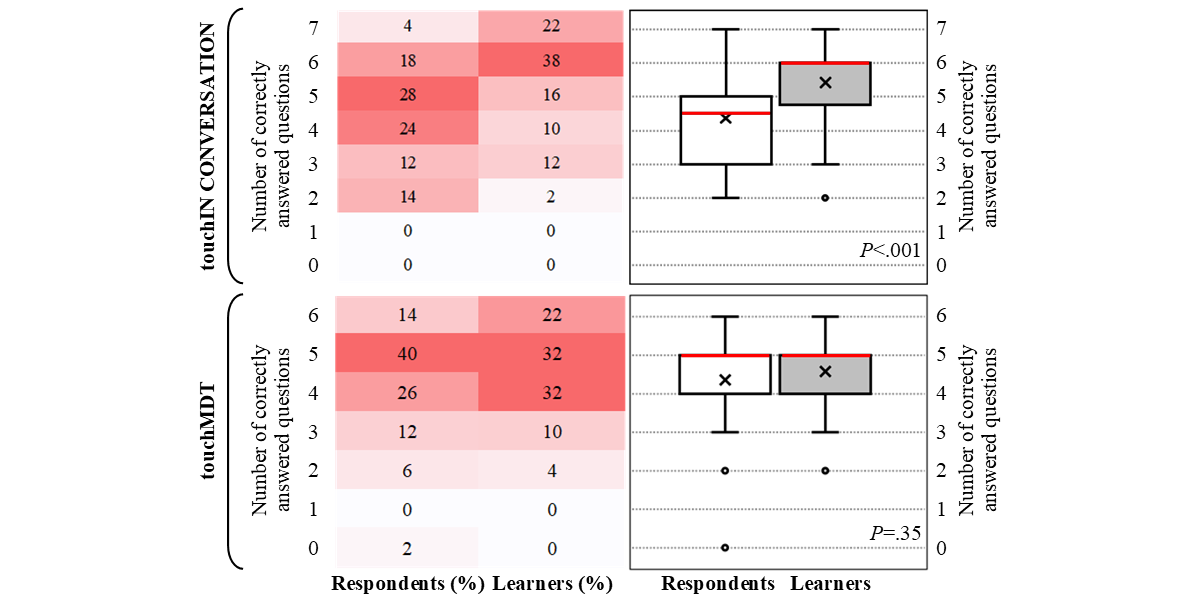
Summary of the number of correct responses for the level 3 and level 4 outcome questionnaires before and after the launch of touchIN CONVERSATION and touchMDT (touch MultiDisciplinary Team).

Before the launch of the touchIN CONVERSATION activity, 22% (11/50) of the respondents answered at least 6 of the 7 questions of the level 3 and level 4 questionnaires correctly, whereas after participating in the activity, this increased to 60% (30/50). There was a significant increase in the average number of correctly answered questions from before to after the activity (median, IQR 4.5, 3.0-5.0 vs 6.0, 4.75-6.0; mean, SD 4.36, 1.40 vs 5.42, 1.37; *P*<.001). These results are shown in Figure 1, where heat maps on the left show the proportion of respondents (n=50) and learners (n=50) who answered a specific number of questions correctly, as displayed by colors ranging from white (lowest proportion of respondents and learners) to dark red (highest proportion of respondents and learners). The box-and-whisker plots on the right show the distribution of the number of correctly answered questions by all respondents and learners. In both plots, the horizontal red line within the box indicates the median, the “x” symbol represents the mean, the boxes indicate the IQR, and the vertical lines (whiskers) extend to the range of values, excluding outliers. Outliers are defined as values that fall outside a distance of 1.5× the IQR from the upper and lower quartiles and are represented by empty circles. Respondents and learners were HCPs who completed the pre- and postactivity questionnaires, respectively.

**Figure 2:**
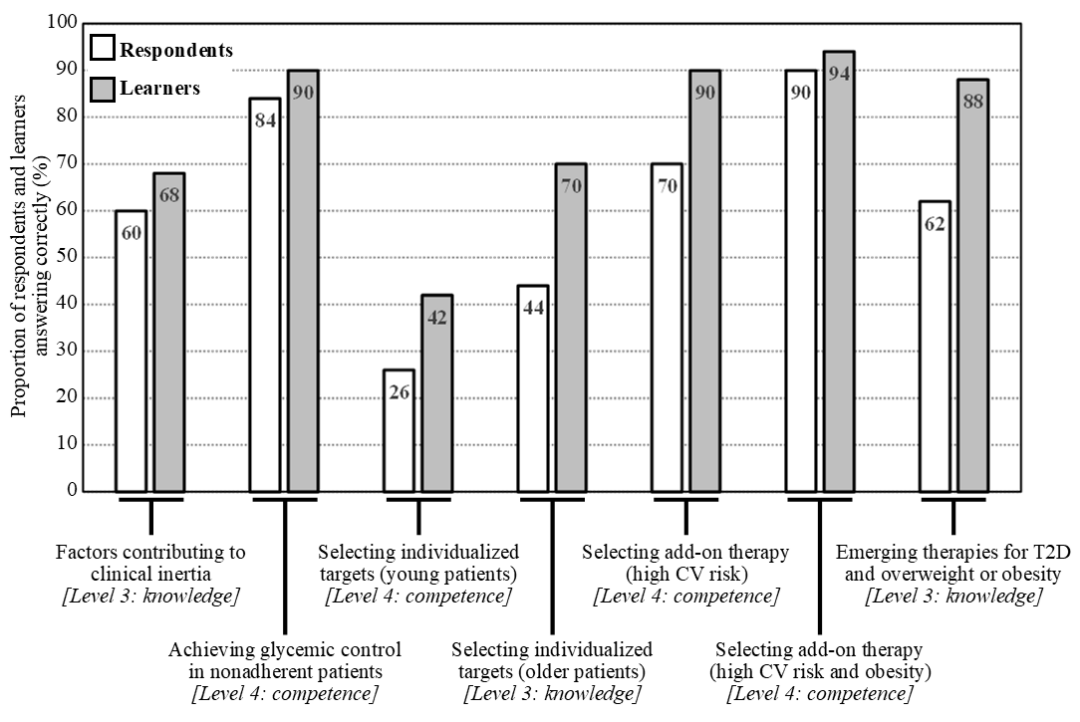
Summary of correct responses for individual topics for the level 3 and level 4 outcome questionnaires before and after the launch of touchIN CONVERSATION. CV: cardiovascular; T2D: type 2 diabetes.

There was also improved knowledge from before to after the activity in the selection of individualized glycemic targets for older patients (22/50, 44% answered correctly before the activity vs 35/50, 70% answered correctly after the activity) and of emerging therapies for T2D and obesity (31/50, 62% vs 44/50, 88%). In addition, improved competence in selecting individualized glycemic targets for younger patients (13/50, 26% vs 21/50, 42%) and in selecting an add-on therapy for patients at a high risk of cardiovascular disease (35/50, 70% vs 45/50, 90%) was observed (Figure 2, where the bar graphs show the percentage of respondents (n=50) and learners (n=50) who answered each question correctly. The numbers within the bars indicate their values. Respondents and learners were HCPs who completed the pre- and postactivity questionnaires, respectively).

**Figure 3:**
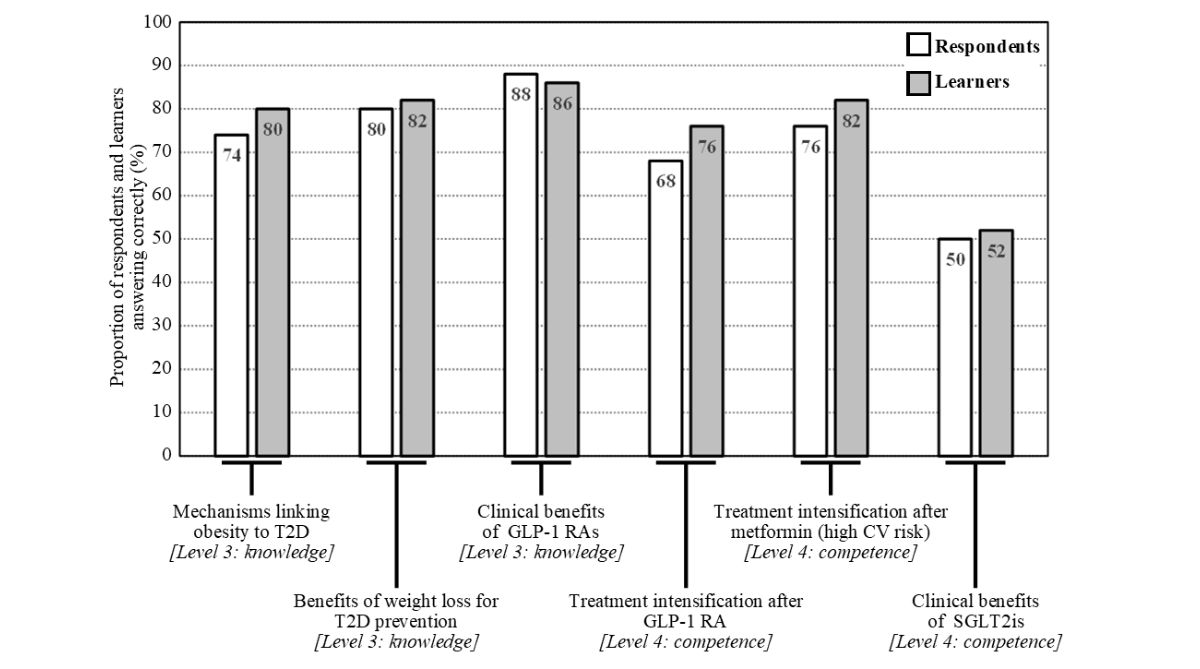
Summary of correct responses for individual topics for the level 3 and level 4 outcome questionnaires before and after the launch of touchMDT. CV: cardiovascular; GLP-1 RA: glucagon-like peptide-1 receptor agonist; SGLT2i: sodium-glucose cotransporter-2 inhibitor; T2D: type 2 diabetes; touchMDT: touch MultiDisciplinary Team.

For the touchMDT, there was no significant increase in the number of correct answers from before to after the launch of the activity (median, IQR 5.0, 4.0-5.0 vs 5.0, 4.0-5.0; mean, SD 4.36, 1.24 vs 4.58, 1.07; *P*=.35; Figure 1). Notably, 80% (40/50) of the respondents answered at least 4 of the 6 questions correctly before the activity was available, indicating high baseline knowledge and competence in this cohort (Figure 1). This increased to 86% (43/50) after the launch, with the greatest improvement observed in competence in treatment intensification after GLP-1 RA treatment (34/50, 68% vs 38/50, 76%; see Figure 3, where the bar graphs show the percentage of respondents (n=50) and learners (n=50) who answered each question correctly. The numbers within the bars indicate their values. Respondents and learners were HCPs who completed the pre- and postactivity questionnaires, respectively).

For both activities, the change in the mean number of questions answered correctly was similar across countries (touchIN CONVERSATION, *P*=.36; touchMDT, *P*=.15; Figures S1 and S2 in Multimedia Appendix 2) and years of experience (touchIN CONVERSATION, *P*=.51; touchMDT, *P*=.90; Figures S1 and S2 in Multimedia Appendix 2). For both activities, respondents specializing in primary care had the lowest mean scores at baseline (touch IN CONVERSATION: 3.62; touchMDT: 4.06; Figures S1 and S2 in Multimedia Appendix 2). Furthermore, a significant difference in the change in the mean number of questions answered correctly was observed between specialties (touchIN CONVERSATION, *P*=.03; touchMDT, *P*=.03), with primary care and diabetes specialists showing the largest increase in scores following the touchIN CONVERSATION and touchMDT activities, respectively (Figures S1 and S2 in Multimedia Appendix 2).

#### Level 5—Performance

**Figure 4:**
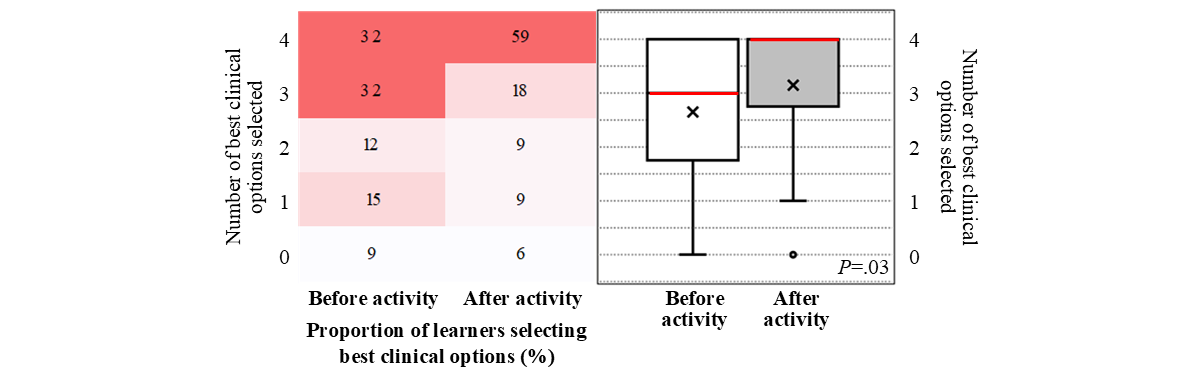
Summary of responses for the level 5 outcome questionnaire before and after the launch of touchIN CONVERSATION and touchMDT (touch MultiDisciplinary Team).

**Figure 5:**
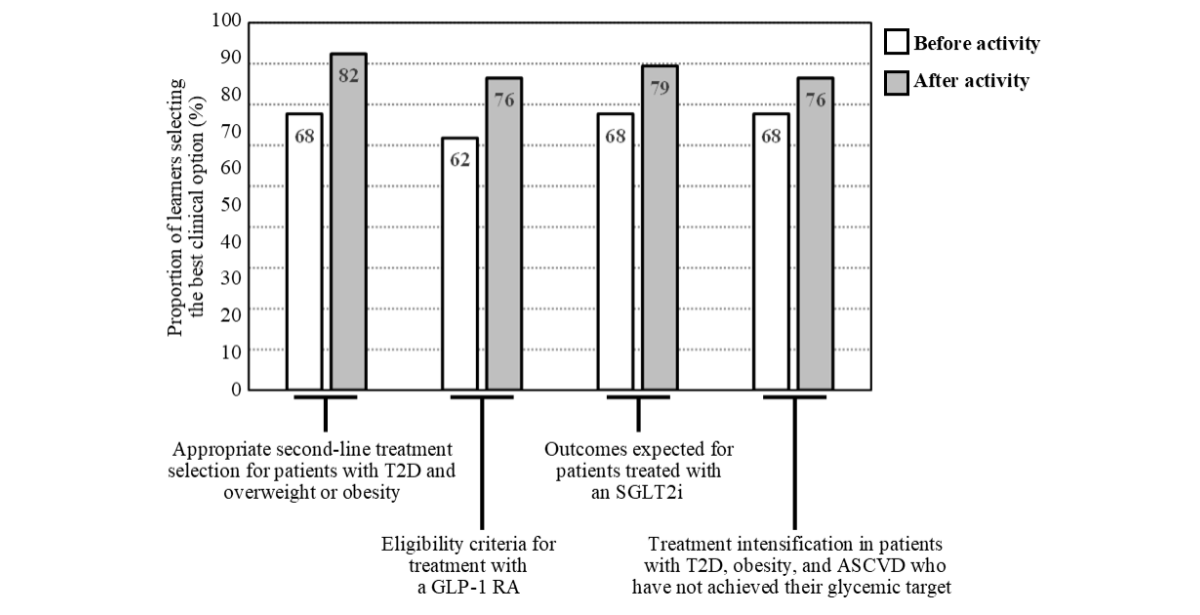
Summary of correct responses for individual topics for the level 5 outcome questionnaire before and after the launch of touchIN CONVERSATION and touchMDT. ASCVD: atherosclerotic cardiovascular disease; GLP-1 RA: glucagon-like peptide-1 receptor agonist; SGLT2i: sodium-glucose cotransporter-2 inhibitor; touchMDT: touch MultiDisciplinary Team.

Before the launch of the touchIN CONVERSATION and touchMDT activities, 32% (11/34) of the respondents selected the answer representing the best clinical option for all 4 questions. This increased to 59% (20/34) after participating in the activities (Figure 4; where the heat map on the left shows the proportion of learners (n=34) who answered a specific number of questions by selecting the best of 4 clinical options before and after viewing the activities, as displayed by colors ranging from white (lowest proportion of respondents and learners) to dark red (highest proportion of respondents and learners). The box-and-whisker plot on the right shows the distribution of the number of questions answered by selecting the best clinical option by all learners before and after viewing the activity. The horizontal red line within the box indicates the median, the “x” symbol represents the mean, the box indicates the IQR, and the vertical lines (whiskers) extend to the range of values, excluding outliers. Outliers are defined as values that fall outside a distance of 1.5× the IQR from the upper and lower quartiles and are represented by empty circles). Overall, a significant increase in the number of best clinical options selected from before to after the activity was observed (median, IQR 3.0, 2.0-4.0 vs 4.0, 2.5-4.0; mean, SD 2.65, 1.32 vs 3.15, 1.26; *P*=.03; Figure 4). Improved performance from before to after the activity was observed for all 4 questions; in particular, questions related to treatment intensification for patients not achieving their glycemic target (23/34, 68% of the respondents gave the best clinical option response before the activity vs 28/34, 82% who gave the best clinical option response after the activity) and eligibility criteria for treatment with a GLP-1 RA (21/34, 62% vs 26/34, 76%; see Figure 5, where the bar graph shows the percentage of learners (n=34) who answered each question by selecting the best clinical option before and after viewing the activities. The numbers within the bars indicate their values).

The change in the mean number of best clinical options selected was similar across the years of experience (*P*=.66; Figure S3 in Multimedia Appendix 2). There was a statistically significant difference in the change in the mean number of the best clinical options selected across different countries (*P*=.03; Figure S3 in Multimedia Appendix 2). Learners from France and Germany showed the lowest and highest mean numbers of best clinical options selected at baseline, respectively; this did not increase following the touchIN CONVERSATION and touchMDT activities for participants from either country (Figure S3 in Multimedia Appendix 2). As in the level 3 and level 4 questionnaires, primary care specialists had a lower mean score at baseline compared with specialists in endocrinology (2.00 vs 2.96), and primary care specialists showed the largest increase in best clinical options selected following the touchIN CONVERSATION and touchMDT activities (*P*=.01; Figure S3 in Multimedia Appendix 2).

### Intention to Change Practice

More than two-thirds (34/50, 68%) of the learners stated that they would change their practice following their participation in touchIN CONVERSATION. Of the remaining learners, 14% (7/50) were uncertain and 18% (9/50) would not make a change. In total, 12% (6/50) of the participants indicated that more education on the subject would be beneficial.

For the touchMDT, more than half (27/50, 54%) of learners stated that they would change their practice following their participation in the activity. A total of 24% (12/50) of the learners were uncertain, mostly owing to practical limitations (7/50, 14%), whereas 22% (11/50) stated that they would not make a change, owing to practical limitations (8/50, 16%). In total, 16% (8/50) of the participants indicated that more education on the subject would be beneficial.

When responding to the level 5 questionnaire, 59% (20/34) of the learners stated that they would make a change to their practice following their participation in the touchIN CONVERSATION and touchMDT activities. Of the remaining learners, 12% (4/34) were uncertain, and 29% (10/34) would not make a change. In total, 18% (6/34) felt that more education would be required, and 21% (7/34) noted that practical limitations would affect their ability to change their practices.

### Identification of Remaining Educational Gaps

Two educational gaps were identified from the questions that were frequently answered incorrectly in the level 3 and level 4 questionnaires: (1) selecting individualized glycemic targets in younger patients and (2) communicating the benefits of SGLT2i therapies. Questions on the first topic were answered correctly by only 42% (21/50) of the learners after participating in the touchIN CONVERSATION activity (Figure 2), and questions on the second topic were answered correctly by only 52% (26/50) of the learners after participating in the touchMDT (Figure 3).

The 3 most important unmet educational needs identified by touchIN CONVERSATION and touchMDT learners after the activity, in response to the question, “What do you think is the most important unmet educational need in this therapy area?” in the level 2 to level 5 questionnaires, are shown in Table 2.

**Table 2:** Unmet educational needs identified by the touchIN CONVERSATION and touch MultiDisciplinary Team (touchMDT) learners^a^.

touchIN CONVERSATION (level 2 to level 4 questionnaires)	touchMDT (level 2 to level 4 questionnaires)	touchMDT and touchIN CONVERSATION (level 5 questionnaire)
1. Use of time-in-range metrics in continuous glucose monitoring to help optimize glycemic control	1. Strategies for achieving sustained weight loss in patients with T2D^b^ and obesity	1. Efficacy data for emerging antihyperglycemic agents for type 2 diabetes and obesity and their use in clinical practice
2. Managing treatment regimens to avoid hypoglycemia in patients with T2D	2. Understanding the data from cardiovascular and renal outcomes trials for antihyperglycemic medications in patients with T2D at high cardiovascular or renal risk	2. Managing the side effects of antihyperglycemic medications in patients with T2D and obesity
3. Efficacy data for emerging antihyperglycemic agents for T2D and their use in clinical practice	3. Use of time-in-range metrics in continuous glucose monitoring to help optimize glycemic control	3. Managing treatment regimens to avoid hypoglycemia in patients with T2D

^a^The top 3 unmet educational needs are shown, as identified by learners who completed the level 2 to level 4 and level 5 questionnaires following the launch of the touchIN CONVERSATION and touchMDT activities. Learners were required to rank 4 predefined, potential educational gaps in response to the question “What do you think is the most important unmet educational need in this therapy area?”

^b^T2D: type 2 diabetes.

## Discussion

### Principal Findings

This study evaluated outcomes following 2 faculty-led, CME-accredited, web-based educational activities on the management of patients with T2D and demonstrated that HCPs expressed high levels of satisfaction and improvements in their knowledge and competence, as well as self-reported performance in T2D management. By the 6- to 7-month postlaunch time point, each activity had been viewed by a global audience of approximately 6000 participants, of which most were specialist physicians. Learners’ satisfaction levels with the educational activities were high, and they considered them to be relevant to clinical practice, meet the stated learning objectives, and impact their future management strategies.

In the touchIN CONVERSATION activity, learners successfully improved their knowledge, competence, and performance, and the benefits of setting individualized glycemic targets were identified as key future educational needs. In addition, most learners confirmed that they would change their practice in response to participation, highlighting the clinical value of the activity. Although an improvement in self-reported performance after participating in the touchMDT activity was reported, no significant increase in knowledge and competence was observed, and fewer learners indicated an intention to change practice compared with the touchIN CONVERSATION activity. This may reflect the relatively high baseline knowledge and competence levels observed in this cohort. However, competence in advising patients on the clinical benefits of SGLT2i therapy was relatively low, with little improvement observed after the activity. This may suggest a requirement for further education or may reflect a bias based on clinical experience with this treatment class. The high baseline scores and subsequent lack of significant increases in knowledge and competence following the touchMDT may also indicate that the activity successfully consolidated the knowledge and competence gained from the earlier touchIN CONVERSATION, which addressed, in part, similar topics. Thus, it is possible that the respondents of the level 3 and level 4 questionnaires for the touchMDT partially overlapped with the learners from the touchIN CONVERSATION, as the target audience and geographies were identical, and participants were reached through the same channels. This interpretation is consistent with the concept of spaced learning, according to which, re-exposing learners to information over time using temporal intervals results in more effective retention of information than if it was all provided at once [40].

Improvements in knowledge and competence were similar across countries and years of experience, indicating that education was beneficial for the entire range of HCPs. Some numerical differences were observed between countries for the level 3 and level 4 questionnaires, with improvements in knowledge and competence seen for learners in the United States, but not in France, Germany, Italy, or Spain following the touchMDT activity. When the impact of education on learners’ performance was assessed, there was a statistically significant difference in performance improvement across different countries, although learners from France and Germany showed no increase in the mean number of the best clinical options selected. Interestingly, although the lack of increase in performance in learners from Germany could be attributed to the high mean of the best clinical options selected at baseline, leaving little room for further increase, learners from France did not show any increase in performance despite showing the lowest score at baseline. Because of the small size of the subgroups, we cannot speculate on the potential reasons for this; further studies with larger groups of participants from these countries are required to obtain meaningful insights. Significant differences were also noted between specialties for the level 3, level 4, and level 5 questionnaires, with the largest improvements observed for primary care specialists. Overall, the HCPs in primary care demonstrated the lowest levels of knowledge, competence, and performance before both activities. This highlights the importance of ongoing education to ensure that primary care teams remain up to date with the rapidly evolving treatment and management landscape of T2D. The high participation numbers and satisfaction scores, combined with improvements in knowledge, competence, and performance observed following one or both activities, support the educational approach of (1) precanvassing prospective learners and using specific patient cases to ensure that the activity is immediately relevant to HCPs’ daily practice and (2) using multidisciplinary faculty and real-life patients to deliver the educational activity.

Most learners indicated that they would implement changes in clinical practice because of their educational activities. Nonetheless, a notable proportion of HCPs who participated in the activities stated that they would not change their practices at the current time. Although patient-centered care is increasingly becoming the focus of health care improvement, several barriers still exist that may prevent its application in daily clinical practice and on a larger scale across health care organizations [41]. These barriers include lack of organizational culture shift, flawed communication and leadership strategies, and practical limitations such as recently updated guidelines, accessibility to emerging drugs and practices, and cost-associated factors [41]. In addition, a recent study assessing factors that influence HCPs’ intention to put newly acquired knowledge into practice identified a lack of belief in one’s own capabilities (ie, the belief that one is capable of performing the behavior) among the barriers to adopting changes in clinical practice. The consequences of adopting new clinical behaviors were also cited as a key barrier [42].

The results of this study indicated that several educational needs remain. As indicated by the questions that were frequently answered incorrectly after the activity, there appears to be a need for more education on setting individualized glycemic targets, particularly in younger patients, and a need to further understand the potential benefits of SGLT2i therapies. In addition, learners self-selected several educational needs, including the use of time-in-range metrics for continuous glucose monitoring, strategies to avoid hypoglycemia, how to achieve sustained weight loss, understanding data from cardiorenal outcome trials, efficacy data for emerging therapies, and managing the side effects of antihyperglycemic medications.

### Strengths and Limitations

The strengths of this study were, first, the involvement of prospective learners in the development of the touchIN CONVERSATION and the provision of a multidisciplinary program, the touchMDT, delivered by and for an interdisciplinary team of HCPs. Both aimed to maximize HCP participation, engagement, and satisfaction with the program. Second, the activities were accredited; thus, physicians, physician assistants, and nurses could obtain CME credit through participation in education. Third, there were no barriers to accessing education, with both activities made freely available on the touchENDOCRINOLOGY website. Fourth, the outcome questionnaire data were collected using an independent sample model, and the questionnaire was fielded to a statistically representative sample. All data collection was carried out by an independent third-party vendor.

This study had several limitations. First, self-selection bias must be considered when assessing the impact of educational activities; thus, HCPs who consider their knowledge to be lacking in these topics are more likely to participate than those who consider their knowledge to be up to date. This bias generally affects medical education, irrespective of the format or delivery method used. To mitigate self-selection bias, we used a combination of different channels to reach the HCP target audience for the activities described here: not limited to clinicians actively seeking medical education, but extended to a heterogeneous population of HCPs, including social media subscribers and members of professional societies. Second, the long-term benefits of educational activities remain unknown. In the future, measuring the impact of education over a longer time frame (eg, at 12 and 24 months) may be beneficial, although the treatment paradigm for T2D is relatively fast-moving, and measuring the impact beyond 24 months may not prove insightful owing to changes in clinical practice. Rather, providing updates to the education based on feedback from learners and the results of the outcomes analysis would be more practical and would ensure that HCPs are kept up to date with information that is useful and relevant. Third, aggregated rather than matched data were used for the level 3 and level 4 questionnaires; however, a previous study of CME outcomes indicated that aggregated data are comparable with matched data and are therefore likely to be sufficiently accurate for many program evaluation purposes [43]. Fourth, as with any analysis of this type, subgroup analyses were limited by the small size of the subgroups, and as such, may not be generalizable to a larger population of HCPs. In future studies, a larger sample size may increase the statistical power of subgroup analyses. Fifth, when assessing the intention to change practice because of the education, a proportion of learners indicated practical limitations as an obstacle to applying changes to their daily practice; however, our questionnaire did not probe the nature of these practical limitations, but it would be beneficial to collect this information in future learning activities to assess whether any of these barriers can inform future education. Sixth, level 5 outcomes were measured subjectively (ie, they were based on self-reported performance rather than on observed changes in patient management). In future studies, a similar approach assessing self-reported performance could be paired with objective evaluations, such as the collection of anonymized patient records, to confirm whether self-assessment is predictive of objective improvement in HCPs’ performance and to provide a more detailed understanding of the impact of CME activities on HCPs’ performance and the health status of the population with diabetes.

### Comparison to Prior Work

CME accreditors are placing increasing importance on the measurement of higher-level outcomes following participation in educational activities; however, data on outcomes from web-based CME activities in T2D are limited [44]. Several studies have demonstrated that traditional CME programs for diabetes can lead to improvements in clinical practice and patient outcomes [10,16,17,20]. However, these studies focused on more time-consuming activities, including face-to-face and live educational sessions, which may make it difficult for HCPs to fit into their schedules. In addition, HCP performance and patient outcomes are often assessed using costly and labor-intensive methodologies, such as objective structured clinical examination stations and patient chart reviews, which are not always practical for assessing outcomes from smaller-scale web-based education. The quality of CME is frequently measured using the Moore level of outcome framework, but studies assessing the impact of short web-based CME programs on knowledge, competence, and performance (Moore levels 3, 4, and 5) are limited [37]. There is growing interest in the use of short activities that encourage learner involvement to provide a more convenient and potentially more effective approach to ongoing education for HCPs, as supported by a small but growing body of evidence. For example, the use of short (15-minute) web-based educational sessions resulted in increases in physician knowledge and competence in studies on diabetes [19] and thrombotic thrombocytopenic purpura [45]. Similarly, the value of learner involvement in T2D education has been demonstrated in a randomized controlled trial, which reported that greater improvements in self-reported competence in diabetes management could be achieved through the use of an educational, case-based game compared with a series of face-to-face lectures and group discussions [15]. Furthermore, a pilot study demonstrated that physicians gained confidence and achieved improved performance in test diabetes cases following participation in an hour-long lecture combined with patient cases in a virtual-world setting [18]. The potential benefits of having learners contribute to the development of educational activities were shown in a pilot study, which demonstrated that a web-based educational activity in attention-deficit/hyperactivity disorder cocreated by PCPs was well received by an audience of other PCPs, although no evidence of its efficacy was available [46]. In this study, we further contributed to this growing body of evidence and demonstrated that web-based CME activities, which can be undertaken in short, easy-to-access sessions, can lead to improvements in HCP performance, as measured by a self-reported questionnaire.

Despite the limitations outlined earlier, the overall objectives of this analysis were met: the study demonstrated significant improvements in the knowledge, competence, and performance of HCPs in the management of T2D and obesity following participation in one or both activities; key outstanding educational gaps were identified; and areas for the improvement of educational outcomes assessment were highlighted, which, alongside the knowledge gained on key educational needs, may help to inform future activities that maximize HCP performance and ultimately patient outcomes.

### Conclusions

This study demonstrated that short, case-based, patient-focused, and multidisciplinary team–led CME activities that HCPs can fit into their clinical schedules achieved high levels of satisfaction and improvements in HCP knowledge and competence, along with self-reported performance in T2D management. Ongoing educational needs identified included setting individualized glycemic targets, particularly in younger patients, and the potential benefits of SGLT2i therapies. These educational needs can be used to inform future educational activities in the diabetes HCP community. The activities described in this study reduce barriers to participation in CME activities, as they are convenient and easily accessible to learners and are free to access.

## Appendix

Multimedia Appendix 1 Images of the touchIN CONVERSATION and touch MultiDisciplinary Team activities.

Multimedia Appendix 2 For the touchIN CONVERSATION and touch MultiDisciplinary Team activities: Tables showing educational gaps and learning objectives, and questions included in the level 3, level 4 and level 5 outcome questionnaires; and figures showing a summary of responses to the questionnaires.

## Abbreviations

ACCME: Accreditation Council for Continuing Medical Education
CME: continuing medical education
GLP-1 RA: glucagon-like peptide-1 receptor agonist
HbA_1c_: glycated hemoglobin
HCP: health care professional
PCP: primary care physician
SGLT2i: sodium-glucose cotransporter-2 inhibitor
T2D: type 2 diabetes
touchIME: touch Independent Medical Education
touchMDT: touch MultiDisciplinary Team

## Data Availability

Data will be shared with bona fide researchers submitting a research proposal to touch Independent Medical Education Ltd. Access requests should be submitted to ADN (Alex.Noble@touchime.org). Data will be made available from the study publication date. Any individual participant data will be shared in data sets in a deidentified and anonymized format.
