# Supplementary material for: The Educational Impact of Web-Based, Faculty-Led Continuing Medical Education Programs in Type 2 Diabetes: A Survey Study to Analyze Changes in Knowledge, Competence, and Performance of Health Care Professionals
Source: JMIR Med Educ. 2022 Oct 14;8(4):e40520. doi: 10.2196/40520 (PMC9617189; doi:10.2196/40520)

# **Multimedia Appendix. Images of the touchIN CONVERSATION and touchMDT activities**

## **Figure 1. The touchIN CONVERSATION activity, ‘Achieving individualized glycemic targets in patients with type 2 diabetes: What are the key considerations?’ available at:** [**https://touchendocrinologyime.org/achieving-individualized-glycemic-targets-in-t2d**](https://touchendocrinologyime.org/achieving-individualized-glycemic-targets-in-t2d) **(activity expires 1 October 2023). a) The touchIN CONVERSATION home page. The activity is freely available on the touchENDOCRINOLOGY website; b) An example of the videos: Video 1 - ‘What are the challenges of achieving glycemic control in patients with T2D, and how can they be overcome?’ Individual questions within each section are accessible via the side-bar on the right.**

## **a)**


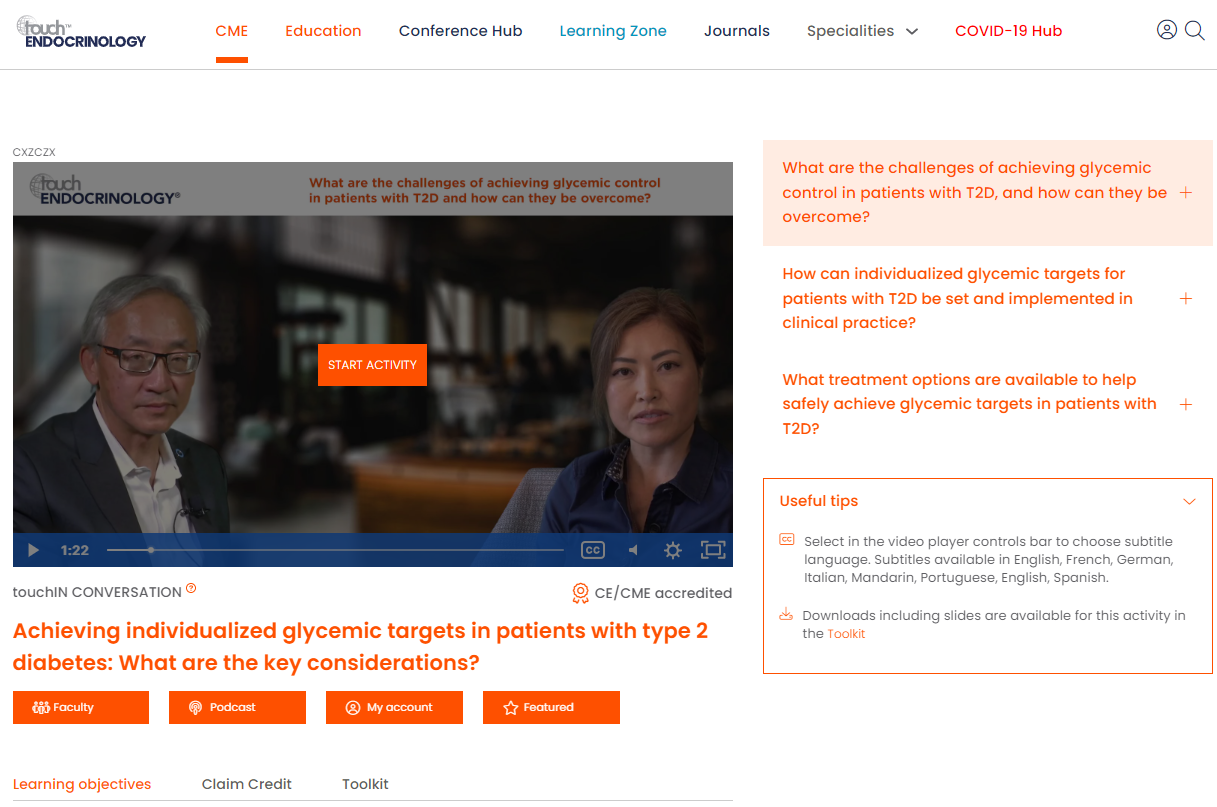


**b)**


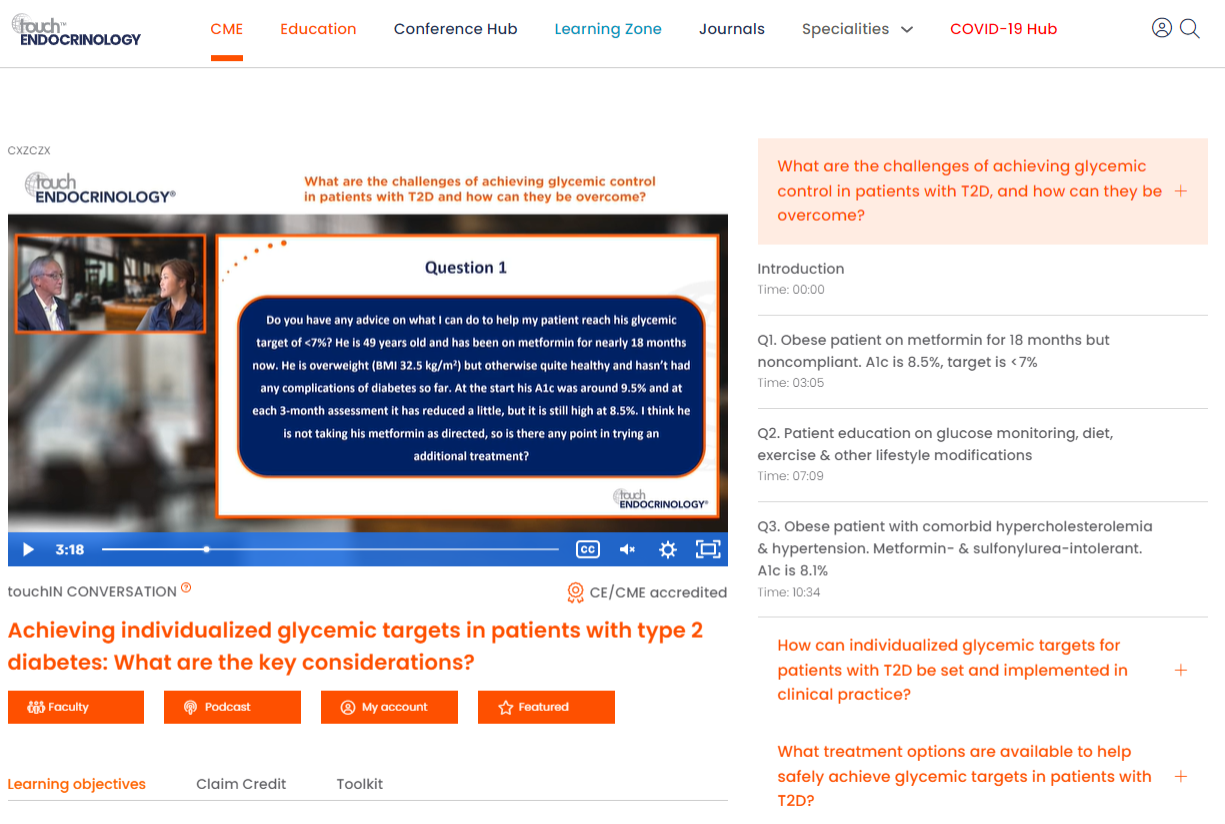


##

## **Figure 2. The touchMDT activity, ‘Antihyperglycemic therapy in the patient with T2D and obesity: A multidisciplinary outlook’ available at:** [**https://touchendocrinologyime.org/antihyperglycemic-therapy-in-t2d-obesity-mdt-outlook**](https://touchendocrinologyime.org/antihyperglycemic-therapy-in-t2d-obesity-mdt-outlook) **(activity expires 14 October 2023). a) The touchMDT home page (‘MDT Hub’). The activity is freely available on the touchENDOCRINOLOGY website; b) An example of the videos: Video 3 - ‘Antihyperglycemic therapy for T2D and obesity: Multidisciplinary perspectives on real-world use’. The individual videos are accessed by selecting a tile on the MDT Hub.**

**a)**


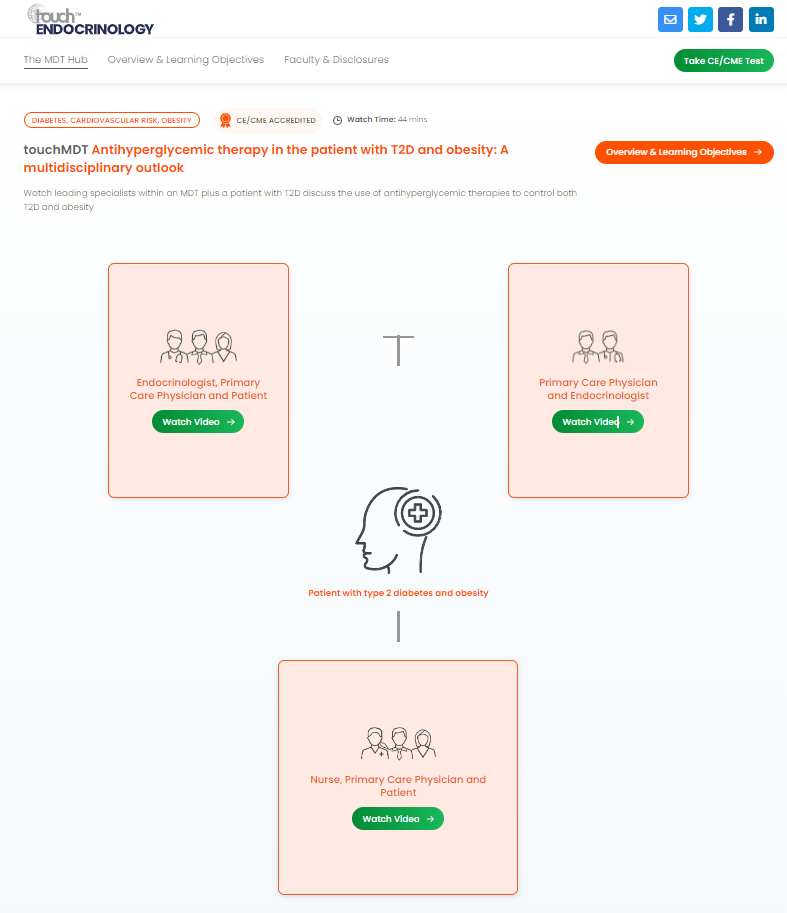


**b)**


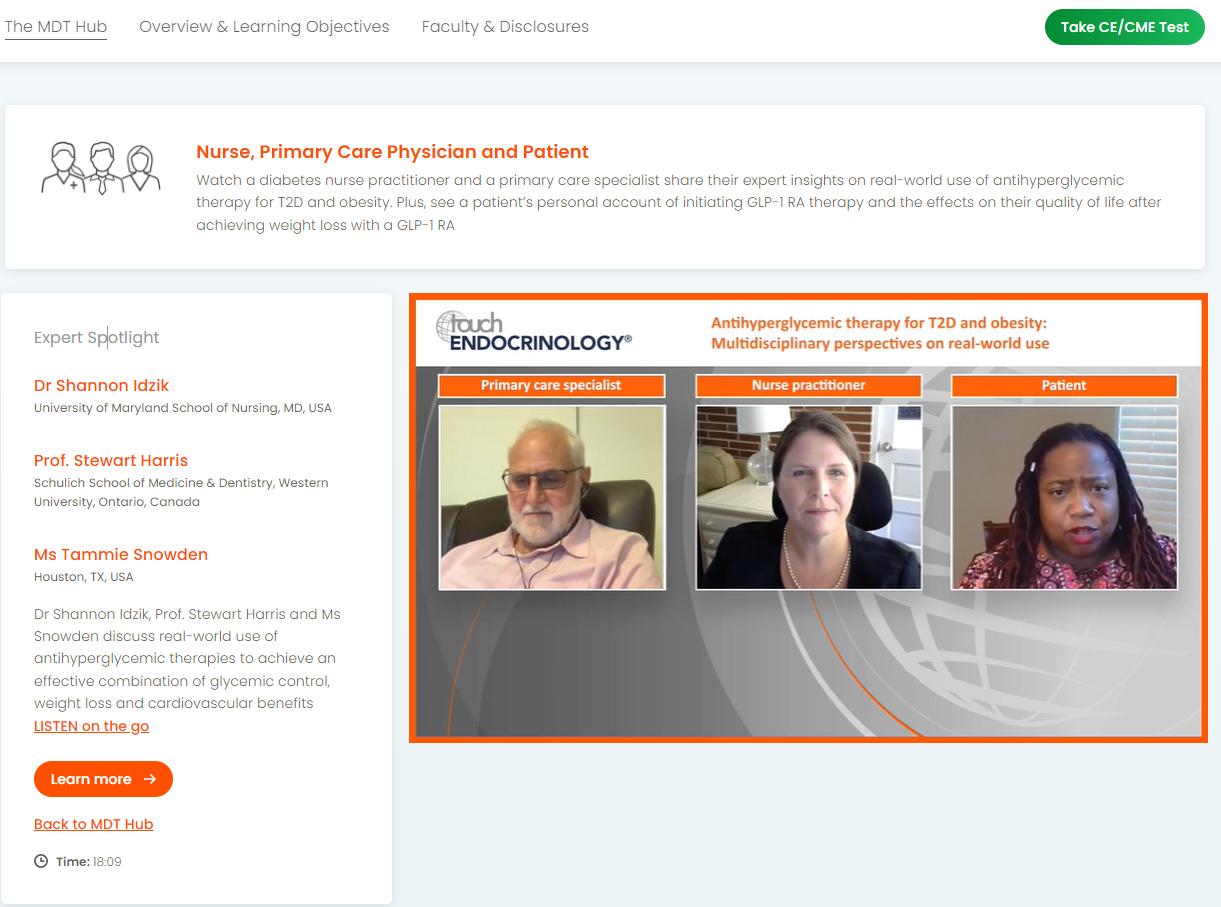

Supplement: Multimedia Appendix 1 [file mededu_v8i4e40520_app1.docx]
