# Supplementary material for: The Educational Impact of Web-Based, Faculty-Led Continuing Medical Education Programs in Type 2 Diabetes: A Survey Study to Analyze Changes in Knowledge, Competence, and Performance of Health Care Professionals
Source: JMIR Med Educ. 2022 Oct 14;8(4):e40520. doi: 10.2196/40520 (PMC9617189; doi:10.2196/40520)

# Multimedia Appendix 2

## **Table S1. Educational gaps and corresponding learning objectives for the touchIN CONVERSATION and touchMDT activities.**

| **touchIN CONVERSATION** |  |
| --- | --- |
| **Educational gaps** | **Learning objectives** |
| Understanding the importance and recognizing the reasons behind the lack of glycemic control in patients with T2D | Evaluate the unmet need regarding achieving glycemic control in patients with T2D and the associated reasons |
| Implementing individualized glycemic targets rather than a one-size-fits-all approach | Decide how to apply individualized glycemic targets according to patient characteristics |
| Selecting appropriate treatments to mitigate the issues that prevent achievement of glycemic control, such as hypoglycemic risk and weight gain | Choose appropriate treatments with properties relevant to the individual patient to help achieve glycemic control |
| **touchMDT** |  |
| **Educational gaps** | **Learning objectives** |
| Recognizing the relationship between obesity and T2D and the need to treat both conditions | Describe the relationship between T2D and obesity |
| Understanding the benefit of antihyperglycemic therapies that are associated with weight loss and reduction in CV risk in patients with obesity and T2D | Predict the beneficial effects of weight loss with a GLP-1 RA-based therapy or SGLT2i on outcomes in patients with T2D and obesity |
| Selecting the appropriate antihyperglycemic therapy based on a patient's characteristics to achieve better control of both T2D and obesity | Perform appropriate selection of antihyperglycemic therapy with weight-loss benefits for patients with T2D and obesity |

## **Table S2. Questions included in the Level 3 and 4 outcomes questionnaire for the touchIN CONVERSATION activity.**

| Questions/multiple choice answers* |
| --- |
| Which of the following are considered to contribute to clinical inertia, and represent barriers to achieving glycemic targets?Proactive care and lack of understanding of new therapiesProactive care and lack of treatment optionsReactive care and lack of treatment optionsReactive care and lack of understanding of new therapies |
| Your patient is a 47-year-old man who has been receiving metformin for 18 months. He has a BMI of 35.5 kg/m^2^ but is otherwise healthy and has not experienced any complications of diabetes. His initial A1c was 10.5%, but it is still high at 8.5%. Upon consultation, he expressed a lack of concern about his obesity and the risks associated with type 2 diabetes, which makes you suspect he may not adhere to his treatment regimen. Which of the following actions would you take?Start him on basal insulin and review him again in 3 monthsRecommend a patient education program and consider starting him on a GLP-1 RAIncrease his metformin dosage and review him again in 3 monthsRecommend a patient education program and consider starting him on a TZD |
| Your patient is a 28-year-old woman who has a BMI of 29 kg/m^2^ and was diagnosed 3 months ago with type 2 diabetes. At the time of diagnosis, she had an A1c of 10.0% and a glucose level of 240 mg/dL. She has tolerated metformin, and at 3-months’ follow-up, her A1c was 9.0%. Based on American Diabetes Association guidelines, what is an acceptable A1c target for you to set?6.5%7.0%7.5%8.0% |
| Which glycemic target would you consider setting for a patient who is over 75 years of age; has had type 2 diabetes for over 20 years; and has cognitive impairment, ASCVD, and CKD?<6.5%<7.0%<7.5%<8.5% |
| Your patient is a 48-year-old woman with HFrEF with an LVEF of 35%. She was recently diagnosed with type 2 diabetes, and prescribed metformin in the first line. She has been identified as having an increased risk of hypoglycemia, and a target of A1c <7.5% was set. After 3 months, her A1c is 8.0%. Which of the following recommended treatment classes would you use as an add-on to metformin?Basal insulinSGLT2iDPP-4iTZD |
| Your patient is a 60-year-old man with a BMI of 30.5 kg/m^2^ and coronary artery disease. His A1c at diagnosis was 10.3%. After 3 months, his A1c is still high at 9.1%. Considering his high BMI and elevated cardiovascular risk, what available treatment class would you consider first to help achieve this target?Basal insulinGLP-1 RA or SGLT2iDPP-4iTZD or SU |
| Pending approval by regulatory bodies, which of the following emerging therapies for type 2 diabetes could you consider for patients who require large reductions in A1c and body weight?TirzepatideImegliminInsulin icodec |

*The correct answer is indicated in bold. Level 3 questions were structured to assess respondents' and learners’ knowledge of guidelines and clinical trial data and how these may be applied in clinical practice, whereas Level 4 questions were structured as patient cases to directly assess the competence of respondents and learners in making the optimal clinical decision. Respondents and learners are defined as healthcare professionals who completed the pre- and post-activity questionnaires, respectively.

A1c, glycated hemoglobin; ASCVD, atherosclerotic cardiovascular disease; CKD, chronic kidney disease; DPP-4i, dipeptidyl peptidase-4 inhibitor; GLP-1 RA, glucagon-like peptide-1 receptor agonist; HFrEF, heart failure with reduced ejection fraction; LVEF, left ventricular ejection fraction; SGLT2i, sodium**-**glucose cotransporter 2 inhibitor; SU, sulfonylurea; TZD, thiazolidinedione.

## **Table S3. Questions included in the Level 3 and 4 outcomes questionnaire for the touchMDT activity.**

| Questions/multiple choice answers* |
| --- |
| One of the key mechanisms linking obesity to the development of insulin resistance, which can ultimately result in type 2 diabetes, is:Obesity suppresses gluconeogenesis, promoting fasting and postprandial hyperglycemiaObesity alters insulin signaling in insulin-sensitive tissues, increasing the ability of cells to take up glucose from the bloodObesity alters insulin signaling in insulin-sensitive tissues, reducing the ability of cells to take up glucose from the bloodIn obese individuals, adipose tissue releases free fatty acids, which stimulates insulin secretion in predisposed individuals |
| 2. What have diabetes prevention studies demonstrated in terms of the minimum weight loss (over a 2-3-year period) that can significantly reduce an overweight person’s risk of developing type 2 diabetes?1 kg (2 lbs)4–5 kg (9-11 lbs)12-15 kg (26-33 lbs)20 kg or more (44 lbs) |
| 3. Your patient is a 54-year-old man, recently diagnosed with type 2 diabetes, with a BMI of 32.5 kg/m^2^. He has been on metformin for 18 months and at his latest visit, his A1c is 8.3%. You decide to prescribe the GLP-1 RA s.c. semaglutide once weekly as an add-on to metformin, but your patient is not familiar with this agent and asks about the main clinical benefits of the treatment class. What would you advise?GLP-1 RAs can substantially lower your body weight, unlike other injectables such as insulin, but only if taken for 12 months or moreGLP-1 RAs will not lower A1c as well as other injectables such as insulin, but can lower your body weight, thereby reducing your risk of CKDGLP-1 RAs are weight neutral, unlike other injectables such as insulin, and maintaining a steady weight can help with achieving A1c goalsGLP-1 RAs can substantially lower your A1c and body weight versus other injectables such as insulin, and weight loss can help with achieving A1c goals |
| 4. After 9 months on the GLP-1 RA semaglutide 1.0 mg, your 54-year-old patient has achieved an A1c of 7.1% and weight loss of 4.8 kg but is still not at his glycemic goal of 6.5%, and his BMI remains >30 kg/m^2^. Which treatment modification would you consider next to help further lower his A1c and body weight?Add a DPP-4iSwitch to a DPP-4iAdd an SGLT2i or basal insulinSwitch to an SGLT2i plus DPP-4i |
| 5. Your patient is a 57-year-old woman with a BMI of 31.0 kg/m^2^ and coronary artery disease. She has been taking metformin and made lifestyle modifications to control her A1c, but at her latest visit, her A1c was still higher than her individualized glycemic target (<7%) at 8.8%. What would you consider next to intensify treatment?Add a GLP-1 RA or SGLT2iSwitch to a GLP-1 RA plus SGLT2i in combination Add basal insulinAdd a TZD or SU |
| 6. Your patient is a 60-year-old woman with type 2 diabetes. She has a BMI of 30.5 kg/m^2^ and was recently diagnosed with stage 3a CKD. She has been taking metformin for 2 years, but at her latest visit, her A1c was 9.0%. When discussing treatment intensification, the patient mentions having heard about an agent called canagliflozin (an SGLT2i) and asks whether it is suitable for her. What do you advise?SGLT2is are appropriate for weight loss, and can reduce the risk of myocardial infarction and stroke in patients with CKDSGLT2is are appropriate for weight loss, and can improve renal outcomes in patients with CKDSGLT2is are not appropriate for weight loss, but can improve renal outcomes in patients with CKDSGLT2is are appropriate for weight loss, but are not suitable for patients with CKD |

*The correct answer is indicated in bold. Level 3 questions were structured to assess respondents' and learners’ knowledge of guidelines and clinical trial data and how these may be applied in clinical practice, whereas Level 4 questions were structured as patient cases to directly assess the competence of respondents and learners in making the optimal clinical decision. Respondents and learners are defined as healthcare professionals who completed the pre- and post-activity questionnaires, respectively.

A1c, glycated hemoglobin; CKD, chronic kidney disease; DPP-4i, dipeptidyl peptidase-4 inhibitor; GLP-1 RA, glucagon-like peptide-1 receptor agonist; s.c., subcutaneous; SGLT2i, sodium**-**glucose cotransporter 2 inhibitor; SU, sulfonylurea; touchMDT, touch MultiDisciplinary Team; TZD, thiazolidinedione.

## **Table S4. Questions included in the Level 5 outcomes questionnaire for the touchIN CONVERSATION and touchMDT activities.**

| Questions/multiple choice answers* |
| --- |
| Which of the following treatment classes do you use most often in the second-line setting for your patients with type 2 diabetes and overweight/obesity, who do not have ASCVD, CKD or HF, and who have not achieved their glycemic target with metformin?TZD or SUDPP-4iGLP-1 RA or SGLT2i Basal insulin |
| 2. Which patient characteristics or criteria do you use to decide whether your patient with type 2 diabetes is eligible for treatment with a GLP-1 RA (where cost is not a major issue)?Need to promote weight loss and patient is at high risk of HFNeed to minimize weight gain, but DPP-4i use is contraindicatedNeed to promote weight loss and/or to minimize hypoglycemia, but DPP-4i use is contraindicatedNeed to promote weight loss and/or to minimize hypoglycemia and/or patient is at high risk of ASCVD or CKD |
| 3. If your patient with type 2 diabetes, obesity and HF is being treated with the SGLT2i canagliflozin, which outcomes do you advise your patient to typically expect as a result of the treatment?Low to moderate weight loss, reduced A1c, lower risk of HF-related hospitalizationA high degree of weight loss, reduced A1c, lower risk of HF-related hospitalizationNo change in weight, reduced A1c, lower risk of HF-related hospitalization and strokeA high degree of weight loss, reduced A1c, lower risk of stroke |
| 4. In your patients with type 2 diabetes, obesity and established ASCVD, currently on metformin plus a GLP-1 RA, which treatment class do you most often use next to intensify treatment, if their A1c remains above their individualized target?TZD or SUSGLT2iDPP-4iBasal insulin |

*The best clinical option is indicated in bold. The Level 5 questionnaire was designed to mainly assess learning objective 3 (treatment choices to achieve glycemic control) for both activities and learning objective 2 ( beneficial effects of weight loss with GLP-1 RA or SGLT2i therapy) for touchMDT.

A1c, glycated hemoglobin; ASCVD, atherosclerotic cardiovascular disease; CKD, chronic kidney disease; DPP-4i, dipeptidyl peptidase-4 inhibitor; GLP-1 RA, glucagon-like peptide-1 receptor agonist; HF, heart failure; SGLT2i, sodium-glucose cotransporter 2 inhibitor; SU, sulfonylurea; touchMDT, touch MultiDisciplinary Team; TZD, thiazolidinedione.

## **Figure S1. Summary of correct responses for the Level 3 and 4 outcomes questionnaire before and after the launch of touchIN CONVERSATION by country (A), level of experience (B), and specialty (C) of the respondents and learners.**

Box-and-whiskers plots show the distribution of the number of correctly answered questions by all respondents and learners. In all plots, the horizontal red line within the box indicates the median, the “x” symbol represents the mean, the boxes indicate the IQR, and the vertical lines (whiskers) extend to the range of values, excluding outliers. Outliers are defined as values that fall outside a distance of 1.5 times the IQR from the upper and lower quartiles, and are represented by empty circles. Respondents and learners are defined as healthcare professionals who completed the pre- and post-activity questionnaires, respectively.

## **
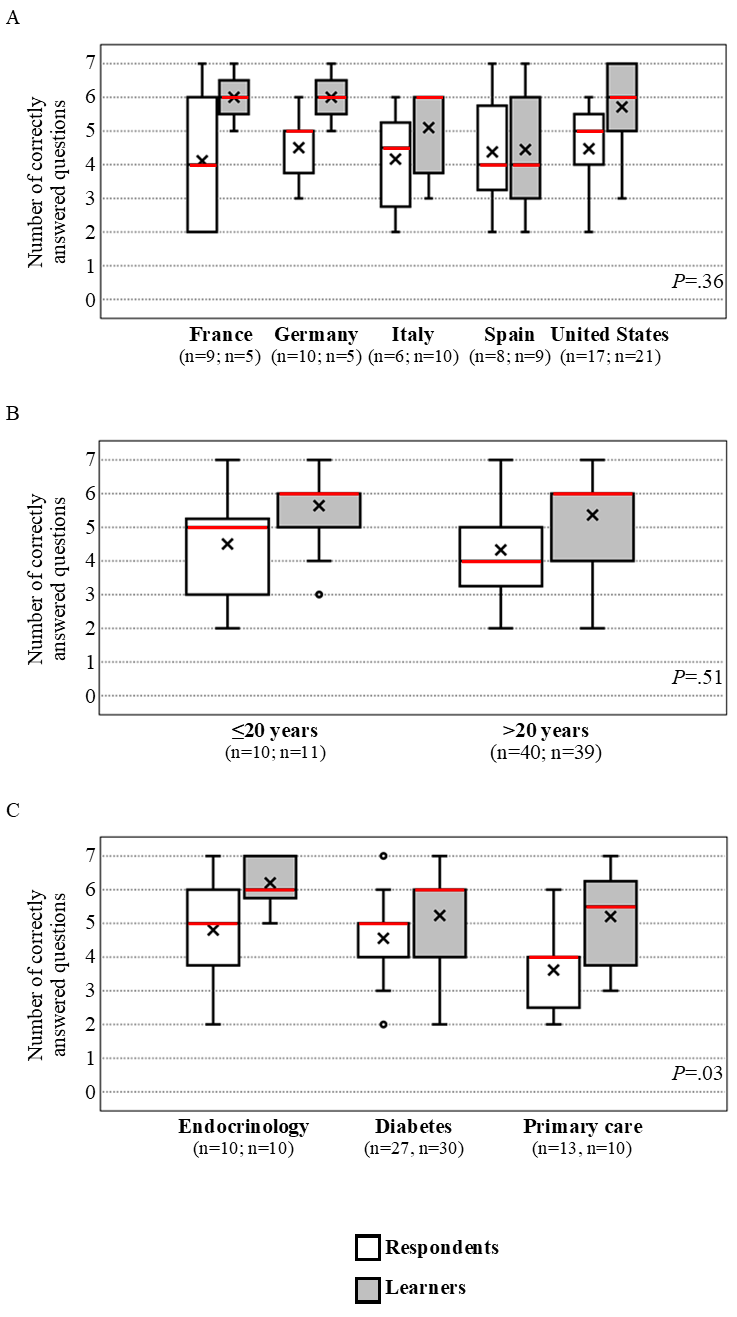
**

## **Figure S2. Summary of correct responses for the Level 3 and 4 outcomes questionnaire before and after the launch of the touchMDT activity by country (A), level of experience (B), and specialty (C) of the respondents and learners.**

Box-and-whiskers plots show the distribution of the number of correctly answered questions by all respondents and learners. In all plots, the horizontal red line within the box indicates the median, the “x” symbol represents the mean, the boxes indicate the IQR, and the vertical lines (whiskers) extend to the range of values, excluding outliers. Outliers are defined as values that fall outside a distance of 1.5 times the IQR from the upper and lower quartiles, and are represented by empty circles. Respondents and learners are defined as healthcare professionals who completed the pre- and post-activity questionnaires, respectively.

touchMDT, touch MultiDisciplinary Team.


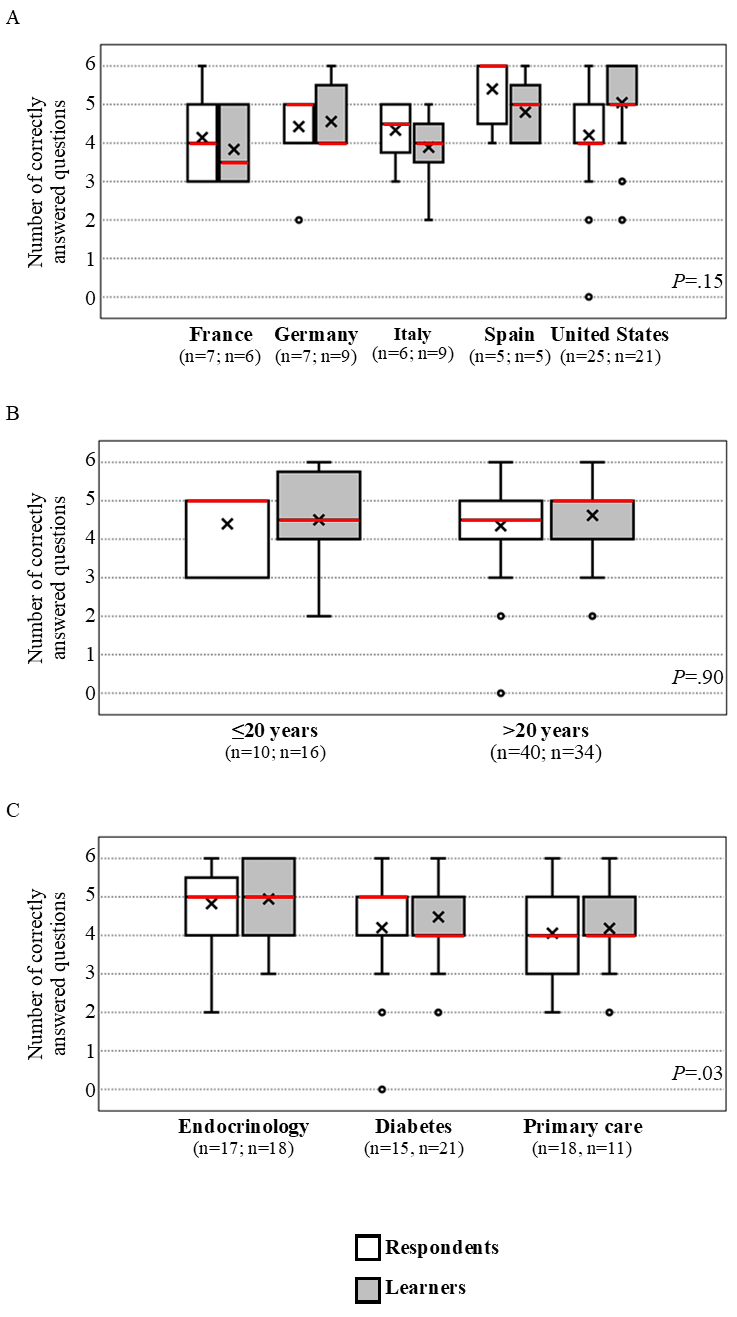


## **Figure S3. Summary of responses for the Level 5 outcomes questionnaire before and after the launch of touchIN CONVERSATION and touchMDT by country (A), level of experience specialty (B), and specialty (C) of the respondents and learners.**

Box-and-whiskers plots show the distribution of the number of questions answered by selecting the best clinical option by all respondents and learners. In all plots, the horizontal red line within the box indicates the median, the “x” symbol represents the mean, the boxes indicate the IQR, and the vertical lines (whiskers) extend to the range of values, excluding outliers. Outliers are defined as values that fall outside a distance of 1.5 times the IQR from the upper and lower quartiles, and are represented by empty circles. Respondents and learners are defined as healthcare professionals who completed the pre- and post-activity questionnaires, respectively.

touchMDT, touch MultiDisciplinary Team.

#
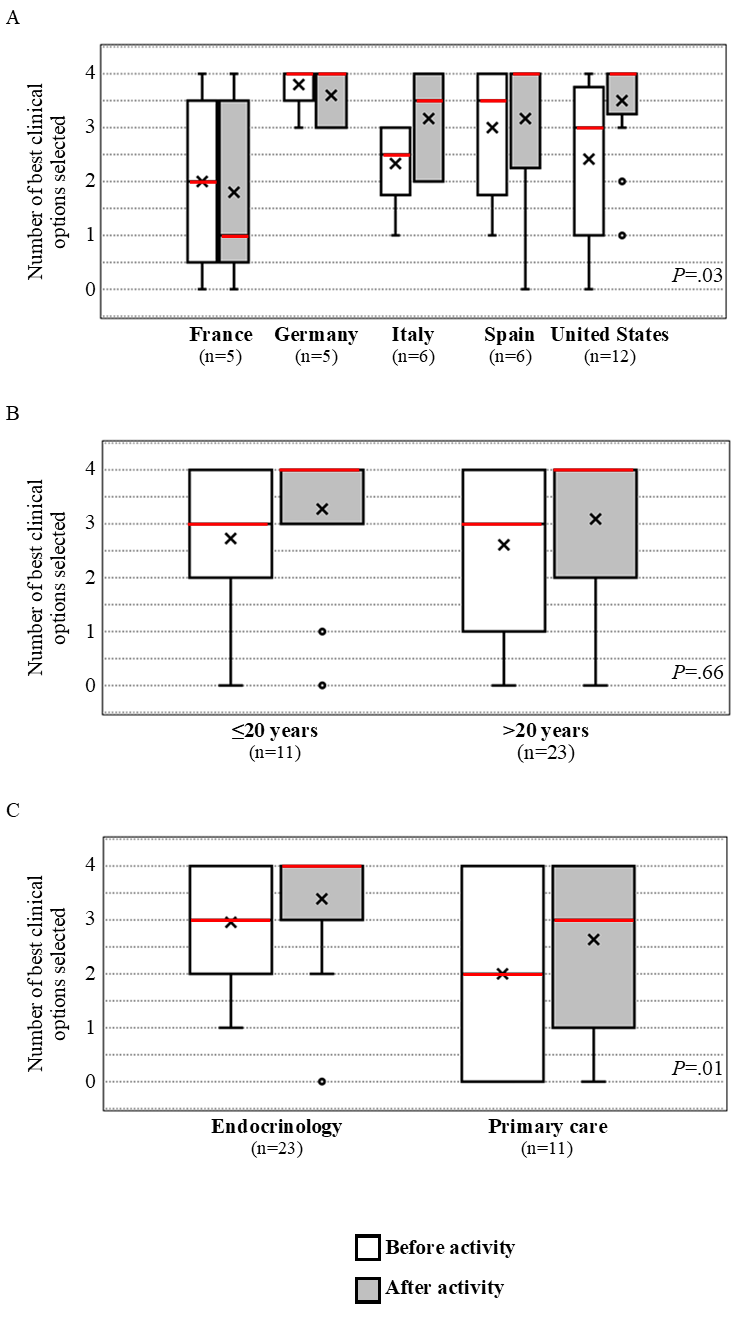

Supplement: Multimedia Appendix 2 [file mededu_v8i4e40520_app2.docx]
